# Pigmented Villonodular Synovitis of the Thoracic Vertebra Presenting with Progressive Spastic Paraparesis

**DOI:** 10.1155/2013/870324

**Published:** 2013-09-15

**Authors:** Mustafa Celiktas, Mehmet Ozan Asik, Yurdal Gezercan, Mahir Gulsen

**Affiliations:** ^1^Ortopedia Private Hospital, Orthopaedics and Traumatology Clinic, 01130 Adana, Turkey; ^2^Hospital Park Darica Hastanesi, Emek Mah, Fatih Sultan Mehmet Cad, No. 131, Bayramoglu Darıca/Kocaeli, Turkey; ^3^Adana Numune Education and Research Hospital, Neurosurgery Clinic, 01240 Adana, Turkey

## Abstract

Pigmented villonodular synovitis (PVNS) is a proliferative benign lesion originating from the synovium and commonly affects large joints of the extremities. PVNS can arise from any synovium in the whole body and rarely affects the zygapophyseal joints of the spine. Spinal PVNS is diagnosed mostly after resection of the mass. In our case we present a 22-year-old male patient showing progressive spastic paraparesis with insidious onset of back pain and difficulty of walking in a relatively short period of 1 month. After gross excision of the mass, diagnosis was established through histopathology. Two years of follow-up period reveals complete resolution of the patient's complaints and no recurrence on radiologic images.

## 1. Introduction

Pigmented villonodular synovitis (PVNS) is a benign proliferative and locally aggressive tumor of the synovium [1, 2]. PVNS may occur in a localized or diffuse form. The localized form is identical histologically to giant cell tumor of tendon sheath. The diffuse form also appears to be identical histologically to the localized form but involves the entire synovium. It commonly affects the synovium of the large joints of the extremities such as knee, hip, and shoulder and theoretically may arise from any synovium in the body [3, 4]. PVNS is characterized as villous or nodular proliferation of the synovial tissue and due to presence of hemosiderin pigment presents yellow to brownish color on gross appearance [4–6]. Only rarely does PVNS affect the axial skeleton, where it arises from the vertebral articular facet joint. Their occurrence in the thoracic spine is a very rare entity; nevertheless, it should be considered in the differential diagnosis [6]. The tumor represents itself with back pain and acute progressive neurological deficit [4–7]. Treatment of the PVNS includes gross resection of the mass and close followup for recurrences. To our knowledge only 54 articles related with PVNS in the spine published in the English literature and most of them are case reports [7]. In this report acute spastic paraparesis with one month of back pain history and PVNS of thoracic 7th vertebra are reported.

## 2. Case Report

**Figure 1:**
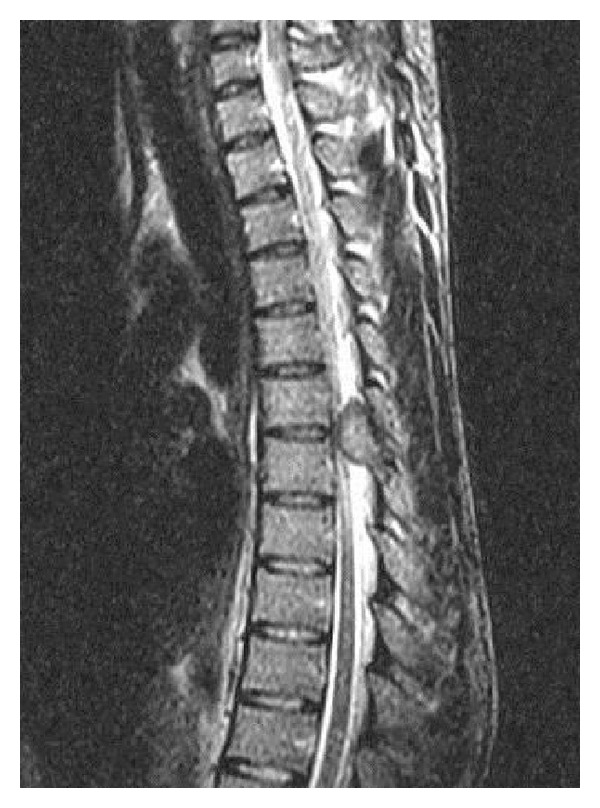
Sagittal T2-weighted images show expansive mass lesion involving the posterior elements of T6 and T7 vertebrae.

**Figure 2:**
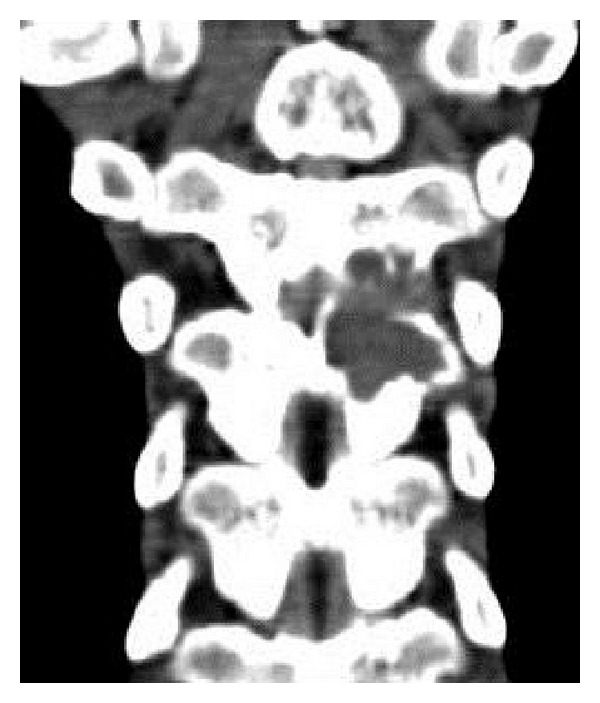
Coronal reformatted CT: eroding areas on the posterior elements of T6 and T7 vertebrae.

**Figure 3:**
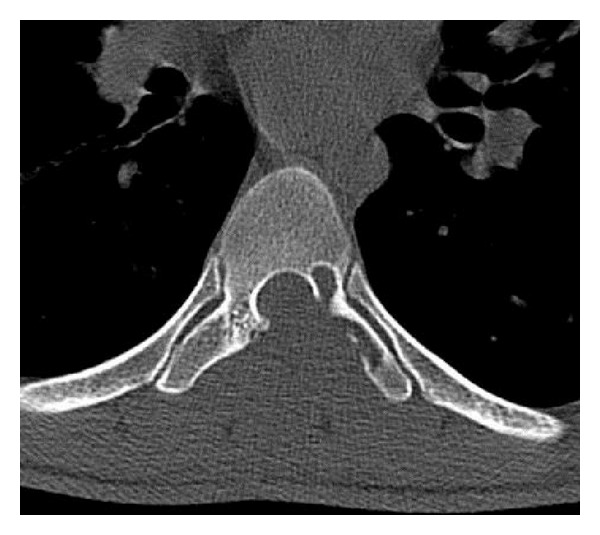
Axial CT scan showing soft tissue mass eroding through lamina and encroaching on thecal sac. There are no foci of calcifications.

A 22-year-old male was admitted to our hospital with a short history of back pain with progressive difficulty in walking. The patient had spasticity in both lower extremities and after hospitalization neurology progressed to paraplegia. Powers of the lower limbs got worse to grade 2-3. Routine hematologic investigations were within normal ranges, and there was no history of trauma, family, and neurological diseases. Immediate MRI and CT scans revealed a tumorous mass that originated from the posterior elements of thoracic 7th vertebra protruding through the spinal cord canal (Figures 1, 2, and 3). After initiating medical treatment (including methylprednisolone 5.4 mg/kg/h for 24 hours and anti-inflammatory drugs), urgent surgery was performed. 

**Figure 4:**
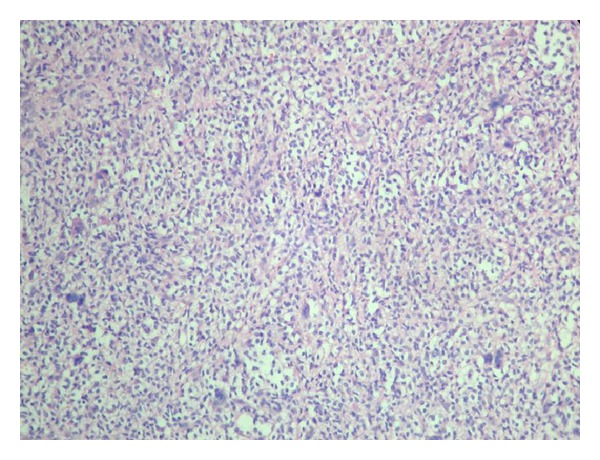
HE ×20 histiocytic and mononuclear cells, multinuclear giant cells.

**Figure 5:**
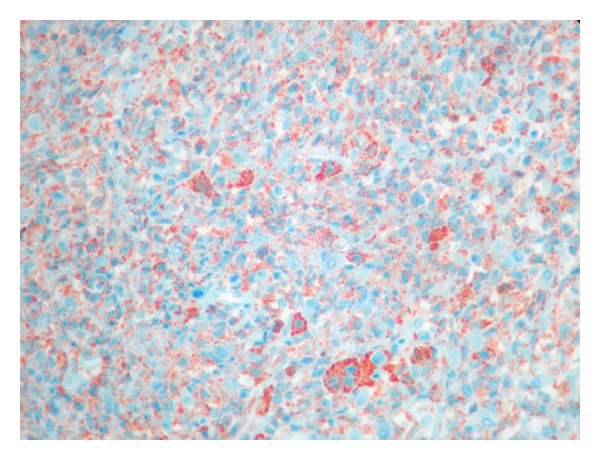
Immunohistochemistry ×40 CD68 positivity in mononuclear, histiocytic, and multinuclear giant cells.

The patient was positioned prone, and a midline incision through T6 to T8 was performed. Thoracic 7th vertebra was exposed, and a total laminectomy and gross excision of the mass were performed. The tumor was avascular and dark brown grossly. Histopathological report of a macroscopically 5 × 4 × 1 cm yellow brown soft tissue after staining with CD 68 revealed mononuclear and multinuclear giant cells with a definitive diagnosis of pigmented villonodular synovitis (Figures 4 and 5).

**Figure 6:**
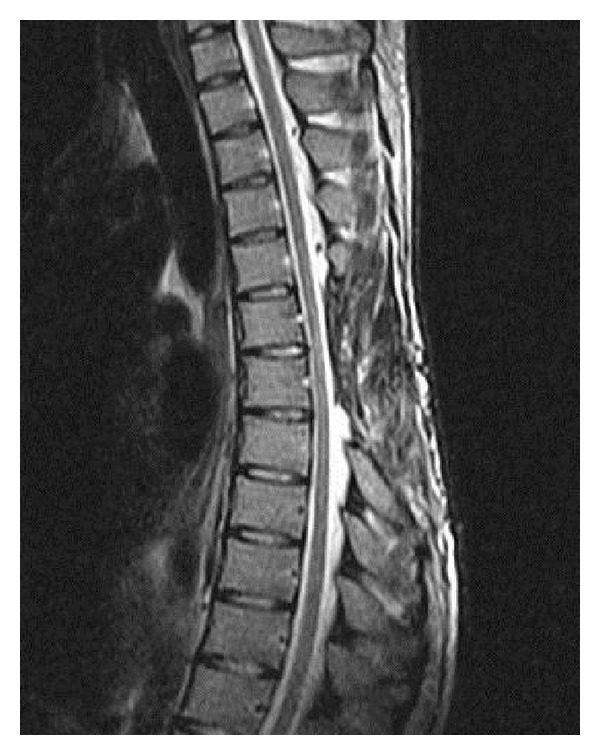
Postoperative 2-year follow-up sagittal MRI showed no expansive mass on the spinal cord and no recurrence.

**Figure 7:**
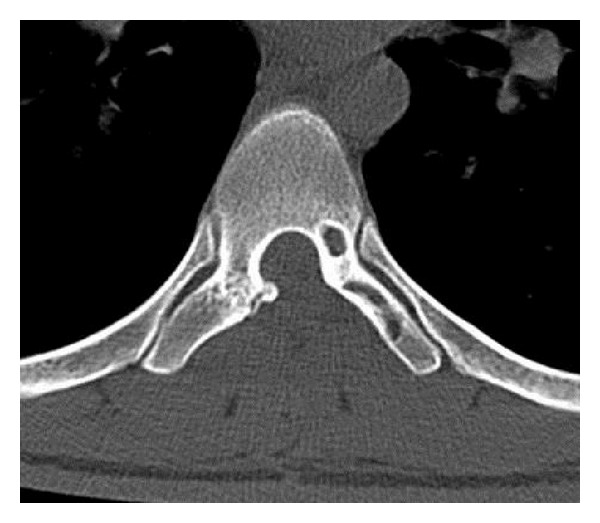
Postoperative 2-year follow-up axial CT scan: the eroded bone areas are in a healing process.

Postoperatively, the patient was followed up for neurological symptoms and after free mobilization was gained he was discharged. He did not receive adjuvant medical therapy. The two-year follow-up period revealed that the patient was without pain and fully reintegrated in his previous job. Radiologic evaluation (MRI and CT scans) showed there was no recurrence of the tumor (Figures 6 and 7).

## 3. Discussion

PVNS arises from the zygapophyseal joint synovium, and its etiology is unknown; tumor growth rate is low, but PVNS usually is locally aggressive. When affecting the spine the first symptom is usually back pain, and progressive neurologic deficit occurs after the spinal cord is compromised [4]. Neurological signs differ according to the level of the tumoral mass. A patient admitted with chronic back pain and acute progression of neurological signs should alert the physician about tumoral and infectious lesions, among which PVNS involves a little proportion [5, 8]. 

In our patient's radiological workup, a lytic, expansile mass having visible borders with 25 × 18 × 33 mm dimensions arising from the T6-7 left facet joint invading transverse process and lamina was visualized. The central canal diminished in size, and spinal cord was compromised. IV contrast material stained the lesion homogeneously. 

Radiological findings of PVNS of the spine are obscure. Aneurismal bone cysts (ABC), osteoid osteoma, and osteoblastoma of the spine should be differentiated, because they all involve the posterior vertebral elements [7]. 

ABC of the spine mimics PVNS as it arises from the neural arch, and does not show matrix mineralization. PVNS differs from ABC by absence of cystic components and does not present liquid-liquid levels, septa are not seen, and also a peripheral rim contrast cannot be visualized [9].

Osteoid osteoma of spine presents itself with painful scoliosis mostly, and affected vertebrae are usually the cervical and lumbosacral vertebrae. Pain at night sometimes diminishes with salicylate consumption [10].

Osteoblastoma of spine is mostly seen in the second and third decades while aneurismal bone cysts presents mostly in the first twenty years of life [10]. PVNS in the literature has been described at all ages of patients ranging from 6 to 84 years for the spinal region. 

On MRI, the intensity of PVNS is not definite, but the demonstration of continuity between the mass and the facet joint helps support the diagnosis of PVNS [9].

PVNS of the spine is seen mostly in cervical and lumbosacral region while in our case a thoracal vertebra involvement was seen. Of the 54 patients reported only 12 of them were reported to involve the thoracic vertebrae [7].

The management options for spinal PVNS include surgery, radiation therapy, and radioisotope infusion. The role of radiation therapy has not been clearly defined. Surgical resection is the primary treatment for this lesion. However, radiotherapy in diffuse lesions may be justified if surgery fails to control the process [3].

## 4. Conclusion

Pigmented villonodular synovitis of the spine is rarely seen, and a histopathological workup is necessary for definitive diagnosis. Acute neurological findings with a relatively short time of symptoms should alert the physician for locally aggressive tumors of the spine, and a radiological workup using MRI and CT may differentiate the lesion before surgery. Age group, clinical findings, absence of matrix mineralization, and homogenous contrast enhancement of the lesion in CT and MRI findings with absence of liquid-liquid levels and relationship of the lesion with facet joint may help surgeon preoperatively. Local recurrences may occur, so gross resection of the tumor and close followup of the patient are mandatory.
